# Hodgkin's disease mortality in Europe.

**DOI:** 10.1038/bjc.1991.388

**Published:** 1991-10

**Authors:** C. La Vecchia, F. Levi, F. Lucchini, S. B. Kaye, P. Boyle

**Affiliations:** Institut universitaire de médecine sociale et préventive, Lausanne, Switzerland.

## Abstract

Trends in mortality from Hodgkin's disease between mid 1950s and the late 1980s have been analysed for 16 western European and seven eastern European countries. In all western countries there were substantial falls in mortality from the late 1960s onwards, for an overall mean decline of 50% in both sexes, although these falls were somewhat larger in Nordic countries (approaching 70% in Denmark and Sweden), and more limited (20 to 30%) in Portugal, Spain and Greece. The reductions in Hodgkin's disease mortality were evident both in younger (under 35) and middle age (35 to 64 years), as well as in children under 15 and, in several countries, in the elderly (above 65), too. They were persistent up to the most recent calendar periods, with no evidence of flattening off. The pattern of trends in Hodgkin's disease mortality was largely different in Eastern Europe. Among seven countries examined, some fall was observed only in Bulgaria and Czechoslovakia, but other countries showed no consistent pattern and there was some increase, too. In absolute terms, the reductions in Hodgkin's disease mortality in Western Europe correspond to the avoidance of over 3,000 deaths per year. This stresses the importance and urgency of improving the availability of currently defined knowledge and resources for treatment of Hodgkin's disease in Eastern Europe.


					
Br. J. Cancer (1991), 64, 723-734                                                                     (?) Macmillan Press Ltd., 1991

Hodgkin's disease mortality in Europe

C. La Vecchia' 2, F. Levi",3, F. Lucchini"3, S.B. Kaye4 &             P. Boyle5

'Institut universitaire de medecine sociale et preventive, Bugnon 17, 1005 Lausanne, Switzerland; 2Istituto de Ricerche

Farmacologiche 'Mario Negri', Via Eritrea 62, 20157 Milano, Italy; 3Registre vaudois des tumeurs, Institut universitaire de
medecine sociale et preventive, Centre Hospitalier Universitaire Vaudois, Falaises 1, 1011 Lausanne, Switzerland;

4CRC Department of Medical Oncology, University of Glasgow, Garscube Estate, Switchback Road, Glasgow G61 IBD, UK;
5SEARCH Programme, Unit of Analytical Epidemiology, IARC, 150 cours Albert-Thomas, 69372 Lyon Cedex 08, France.

Summary Trends in mortality from Hodgkin's disease between mid 1950s and the late 1980s have been
analysed for 16 western European and seven eastern European countries. In all western countries there were
substantial falls in mortality from the late 1960s onwards, for an overall mean decline of 50% in both sexes,
although these falls were somewhat larger in Nordic countries (approaching 70% in Denmark and Sweden),
and more limited (20 to 30%) in Portugal, Spain and Greece. The reductions in Hodgkin's disease mortality
were evident both in younger (under 35) and middle age (35 to 64 years), as well as in children under 15 and,
in several countries, in the elderly (above 65), too. They were persistent up to the most recent calendar periods,
with no evidence of flattening off. The pattern of trends in Hodgkin's disease mortality was largely different in
Eastern Europe. Among seven countries examined, some fall was observed only in Bulgaria and Czechoslo-
vakia, but other countries showed no consistent pattern and there was some increase, too. In absolute terms,
the reductions in Hodgkin's disease mortality in Western Europe correspond to the avoidance of over 3,000
deaths per year. This stresses the importance and urgency of improving the availability of currently defined
knowledge and resources for treatment of Hodgkin's disease in Eastern Europe.

Effective therapies for Hodgkin's disease have been available
for over 30 years (Rosenberg, 1989). These therapeutic
successes have been based on a combination and integration
of better diagnostic methods to evaluate the extent of the
disease (lymphography, diagnostic laparotomy with splenec-
tomy, bone marrow biopsy and computerised tomography),
of technical advancements in radiotherapy (supervoltage
machines and linear accelerators, allowing the delivery of
tumoricidal doses to well identified lymphoid fields) and the
development of combination chemotherapy, particularly
MOPP (mechlorethamine, vincristine, procarbazine, and pred-
nisone) and ABVD (doxorubicin, bleomycin, vinblastine and
dacarbazine) (De Vita et al., 1965, 1970, 1980; Bonadonna,
1982; Rosenberg, 1989). Still, availability and utilisation of
curative treatments is not homogeneous and a summary
tabulation of overall age-standardised mortality from the
disease in various European geographic areas for the period
1978-82 has shown elevated rates in several Central and
Eastern European countries (Yugoslavia, Switzerland,
Czechoslovakia), with a 3- to 5-fold difference compared with
the lower rate areas in France and Denmark (Levi et al.,
1989).

It was decided, therefore, to further investigate trends in
Hodgkin's disease in Europe, and to present in this paper
details of trends in each separate country and selected age
groups.

Materials and methods

Death certification numbers for Hodgkin's disease, stratified
for sex and 5 year age groups for 30 European countries
(excluding the Soviet Union and a few small countries like
Malta, Liechtenstein, etc.), were derived from copies of the
original computer tapes of the World Health Organisation
(WHO) database.

During the calendar period considered (1955-88), four
different Revisions of the International Classification of

Diseases (ICD) were used, but there were no changes in the
definition or coding of the disease between various Revisions.

Estimates of the resident population, generally based on
official Censuses, were obtained from the same WHO data-
bank. From the matrices of certified deaths and resident
populations age-standardised rates (using the World Stan-
dard Population) were computed (Doll & Smith, 1982).
Besides overall age-adjusted rates, four different (age-stan-
dardised) truncated rates (0-14, 15-34, 35-64 and 65 and
over) were chosen for presentation.

In a few countries, data were missing for part of the
calendar period. When a single year was missing within a
quinquennium, numerators and denominators were interpo-
lated linearly from the previous and subsequent calendar
year. No extrapolation was made for missing data at the
beginning or the end of the calendar period considered, or
when data on one or more quinquennia were not available.

For countries covered by national cancer registration
schemes, mortality rates were contrasted with incidence
trends, derived from subsequent volumes of 'Cancer Inci-
dence in Five Continents' (Doll et al., 1966, 1970; Water-
house et al., 1976, 1982; Muir et al., 1988).

Results

Trends in mortality from Hodgkin's disease are presented in
Figure 1 for Western Europe and in Figure 2 for Eastern
Europe in males and females of all ages and in two separate
age groups (0-34 and 35-64 years). Rates for the first
(usually 1955-59) and last (usually 1985-88) calendar
period, together with the corresponding average absolute
number of deaths per year and per cent changes, were further
tabulated in Table I for population of all ages, in Table II
for children under age 15 and in Table III in the oldest age
group (65 years and over).

In all western countries there were substantial declines in
mortality. The declines in all age mortality from Hodgkin's
disease between the late 1950s and the mid 1980s were
around the overall mean of 50% in both sexes (Table I) and
most countries, except for Nordic Countries (and particularly
Denmark and Sweden, whose fall approached 70%), and
Portugal, Spain and Greece (with falls between 20 and 30%).

These declines tended to start between the late 1960s and
the early 1970s in most countries, and were evident both in

Correspondence: C. La Vecchia, Institut universitaire de m&decine
sociale et preventive, Bugnon 17, 1005 Lausanne, Switzerland.
Received 19 March 1991; and in revised form 31 May 1991.

'?" Macmillan Press Ltd., 1991

Br. J. Cancer (I 991), 64, 723 - 734

724    C. LA VECCHIA et al.

MALES

)5         AUSTRIA           E 5

F          _, - A A

0

0  3-
0
0

2

(D

I Cu  1  .

0   55   60  65   70   75  80   85  90     a

Calendar years

0-

BELGIUM
W_

55 60 65 70 75 80 85 90

Calendar years

FEMALES
AUSTRIA

55  60   65   70  75   80   85  90

Calendar years

0F
U)

(D 5 -
E

,.-  4

0
0

0 3

0
0

2     *-i

CD

a)

C  1   *

55      60

BELGIUM

CS S

65  70   75   80  85  90
Calendar years

0

Calendar years

5 -           FINLAND
4-
3-

2 o-0-------O

55  60   65   70   75  80   85  90

Calendar years

(a
0

E
0)

0
0

0
o;
0

0)
T)

4_

Cu
E

0)
a

0
0
0
0
CD

0)
Co

Cu
0)
0

cn
s

DENMARK

4-
3-

2-

1 -

o-o?o?o

55  60  65 70 7  80  85I

55   60  65  70  75  80   85  90

Calendar years

FINLAND

55 60 65 70 75 80 85 90

Calendar years

Figure 1 Trends in mortality from Hodgkin's disease in 16 western European countries from 1955 to 1988. Age-standardised
(world) rates in males and females of all ages (0), at ages 0-34 (@) and 35-64 (M).

la

L 4 -

o
0

6s31

01

CD

E 4-

0
0

0 3-

0

2-

U)

' 1-
-C

4u

co
01

0)

Cu
E

0

U)
0)
..o
co

0)
a

U)
0

0

E

0
0

0

6

0

,r-

a
0

4-c
0)

0

n

I          -  ; I T

u

0----O

i                  I      I     -I-   -I-    - -

cl c_

i        I         I         I         I                  I         I

. -      --                 --        --        ^-       rl?       f%f%

1.

S-1

) -1

I

HODGKIN'S DISEASE MORTALITY IN EUROPE  725

MALES

rCD A 1Kit'%

6 7AN0   8

560   65   70  75  80   85   90

Calendar years

WESTERN GERMANY

5 60 65 70 75 80 85 90

Calendar years

GREECE

5 60 65 70 75 80 85 90

Calendar years

Co

-) 5-

E

D 4

0

O 3-

o
0

? 2-
Co

1 -
.C

-cn

+. 0-

0

i) 5-

4
0)

o 3'
0
6

o 2
Co

r- 2

cn

4) i

.  O.

1
Co

a)0

'5   -
ao

E

01 4'
0
0

ov 3

0

Ir- 2-
(a

Cu  1.

1-

I) 0c

a)
fn

FEMALES
FRANCE

I  5   I5 5  Ig -r  -I

i5   60   65   70   75  80   85   90

Calendar years

WESTERN GERMANY

5     60   65   70  75  80  85   90

_6  6 5  7 0  7 5  8 0  - -   80

Calendar years

GREECE

5  60  65   70  75   80  85   90

Calendar years

aD 5         IRELAND

E

0 4       ,

a

0
0

Co  2 -   -
Cu

o   55 60 65   70  75  80 85 90

Calendar years

Co

0

Cu

E

Cu

0

6
0

L-
0

IRELAND

5.
4.

3.

2
1o

0 -

55  60 65   70   75  80  85 90

Calendar years

lb

u 5
(D

E 4
0

C> 3

co0
0
co

E 2.

CD

1*
co

(D

c

C'
0

2 4-

co

T
-Cu-

-c3

m

2 0-

0
Co

-z 5-
Cu

4-
0

0o 3-
0

2-

Cu

1-
-co
C 5-

L.j

i              I              I              I               .     0        .               I              I

726    C. LA VECCHIA et al.

lc

E 4
0
0

o  3

2

In

in 1*

-o

W 0
CU

U)

(D 5-

E 4

0
0

o   3

0

en 2-

CU

.  1-

W o

CU  0-

a) 5-
o
16

CU 5

E 4-

0
0

,2-

)3
CU

~ 1-
C

0*
a)

MALES
ITALY

-.

5 60   65  70   75  80 85 9o

Calendar years

NETHERLANDS

5  60   65   70   75   80  85   90

Calendar years

NORWAY

55  60   65  70   75   80  85  90

Calendar years

U)
w1

.i 5.
E

'o 4.
0
0

o  3.

0

_ 2
In

in 1*
-C
CU

LI

- 5-

CU

E

4 4-

0
0

o 3-

6
0

--2-

co
w

CU 1-

m o-

wU  5
0

cn

-  5-

CU

E

%  4.

0
0

o 3-
o

0

r-
CU
CU

w
I..

_,
(a

C])

2
1o

0*

FEMALES

ITALY

60

a- 0

60 i5 7'0 i5 80 85 so

Calendar years

NETHERLANDS

5 60 65 707       0  59

5 60 65 70 75 80 85 90

Calendar years

NORWAY

= p

S

. 0   0 ~ S p

55 60 65 70 75 80 85 90

Calendar years

CD

(D  5-

E

E 4-

0
0
0

? 3
0r.-

cs  2 -

1-
0 -

PORTUGAL

n

D 5-
CE

E

CU  4-

0

o  3-

r  2-

in

C

.-o
? o-

I           I       I        I        I

CU
1-
CU

0)

a

1

0-

PORTUGAL

55  60  65  70   75  80  85  90

Calendar years

4-

CU

i  I                     I            I             I            .

i  I

4--

I

I

5

I

4

I                                         -

I            I                      I

55 60   65  70  75   80 85 90

Calendar years

----o

I     I                  I t=l-=:i

HODGKIN'S DISEASE MORTALITY IN EUROPE  727

MALES
SPAIN

o

5  60   65  70  75   80  85  90

Calendar years

SWEDEN

6 60      65  70     75    80    85   9(

Calendar years
5,      SWITZERLAND

4-

3- 1
2 -I
1-  1

55

60  65  70   75  80

Calendar years

85 90

u)

-i 5-
E

1*2 4.
0
0

o 3

0

r? 2

a)
0)

0

0)9
a,

o

'

co 5.
E

0)

4- 4.
0

0:: 3.-
0

0)

o 1-
4-c

co

o )

0 1

Un
a)
Cu
E

0

.  1.

0
0
0
0

4-C
0)
0

FEMALES

SPAIN

5   60  65   70   75  80   85  s0

Calendar years

C-%AIr7rr7II

WVVEDLN

0 --w

55  60   65  70   75   80  85   90

Calendar years

5-
4-

3-
2 -
1 -
0 -

SWITZERLAND

5  60   65   70  75   80  85   90

Calendar years

5-       UNITED KINGDOM
4-

3-

2    o- O - -

0 -

55  60   65   70  75   80  85   90

Calendar years

U)

E

0)

0
0
0
0
U)
0

-
co
0

L-

5.
4.
3 .

UNITED KINGDOM

2

1

.    5 55 za

55  60   65  70      75   80  85  90

Calendar years

ld

W
U)

0) 5-

Cu

o~ q

2-

U) '

0)

.   1 -

4-1

-c

m0)  .

0
cn

(D 5-

E 4
C)

3 -
2 -
1 -
0

-

0

U1)
0)
U.o
4-C
co
Cu

0)
0

n
0)
Cu
E

0
0
0

0
0
0)

0
a

Un
0)
Cu

E

0
0
0
6
0

-

a

0

i

I             I            I              a            I             I              I

I            I                           I

4-

o1 1 I I I I I I

k

0

I

728    C. LA VECCHIA et al.

MALES

5 s          BULGARIA
E 4-

Co

0

0^ 3-

m 1-
0

55  60  65  70   75  80  85 90

Calendar years

CZECHOSLOVAKIA

U,

Q) 5-~
E 4 -

02

s 3

O   55  60  65  70  75   80  85  90

Calendar years

GDR

55

5-
4

3-
2-

1-

55

-U:
p..

60I  65 7  75  80 8  90

60 65 70   75 80 85 9'0

Calendar years

HUNGARY

0 _ _o ...0 --:

o   C        c?

60  65  70   75  80 85   90

Calendar years

0)

-, 5.
E

(D 4.

0
0

oC 3
6
0

2
CD
0)

1'

i 0'

I

FEMALES
BULGARIA

5     60  65  70  75  80  85  90

Calendar years

a,        CZECHOSLOVAKIA

0)

m0-4

E

-*- 4 -

0

0

?:: 3 -

0

0)

T---'2  ,-         1

0)

'O.

o   55 60   65  70  75   80  85  90

Calendar years

CA,

0)

a) 5             GDR

Co

E

) 4

0

0

av 3-
6

0

ns-  1                 o a   o --

L-               0----O -------.

.r                     *     *  -0

0)  55  60  65  70  75   80  85  90

Calendar years

0

co
E
0)

o

0)
o

-c

co

a)

o    55  60   65  70   75   80  85   90

Calendar years

Figure 2 Trends in mortality from Hodgkin's disease in seven eastern European countries from 1955 to 1988. Age-standardised
(world) rates in males and females of all ages (0), at ages 0-34 (0) and 35-64 (U).

2a

0

(D 5-

E 4-

0

CD3C
,-0

-- 2-
0)

CD  1-
o.

0
0
Co

E
0
0
0

0
0

U,

0)

Co
6-M
Co

0
0

, \

i               I               I                                I               I

1--

0

li

I

I

HODGKIN'S DISEASE MORTALITY IN EUROPE  729

MALES
n

a) 5-        POLAND

E 4 -

0
0

0

0)

a) 3?-     .  ._  ..4.
0)

o  55 60 65 70 75 80 85 90

Calendar years

5-
4-
3-
2-

I
1 -
0 -

ROMANIA

55  60   65  70  75   80  85  90

Calendar years

0)

5-5
E

40)4-
0
0

CO3-
6
0
CD
0)

0) 55
a

Co

0)a-

co

E

w0) 4.-
0

o 3

Co

r-  2-

a)

m 0 -

K

0) 55
a

FEMALES
POLAND

u-ua

* - -                       - |

60   65   70   75   80   85  90

Calendar years

ROMANIA

---

560    65  70  75   80  85 90

Calendar years

YUGOSLAVIA

. u~

60  65   70  75  80   85  90

Calendar years

0
0)

E

0)

0

6

Co

0)
0)
co

0

0

5-
4-
3.

2-

1-

0 *

YUGOSLAVIA

A I = I I I I I

55  60   65  70   75   80  85  90

Calendar years

Table I Overall death certification ratesa/100,000 people (and numbers of deaths) from Hodgkin's disease in selected European countries,

1955-59 and 1985-88

Males                                           Females

1955-59           1985-88                       1955-59            1985-88

Rate              Rate          Percent         Rate               Rate          Percent

(No. of deaths    (No. of deaths   difference   (No. of deaths     (No. of deaths   difference
Country (year)               per year)         per year)       in rates      per year)          per year)      in rates
Western Europe

Austria                      2.37 (93)         1.15 (54)         -51          1.66 (79)         0.68 (48)        -59
Belgium                      2.39 (127)        1.18 (40)         -51          1.28 (70)         0.85 (32)        -34
Denmark                      2.35 (58)         0.79 (29)         -66          1.52 (39)         0.33 (15)        -78
Finland                      1.88 (39)         1.01 (30)         -46         0.78 (19)         0.38 (17)         -51
France                       1.62 (387)        0.75 (261)        -54          1.04 (273)        0.36 (152)       -65
W. Germany                   2.08 (572)        0.99 (397)        -52          1.19 (387)        0.57 (321)       -52
Greece (1965-69)             1.86 (87)         1.21 (81)         -35         0.87 (47)          0.58 (42)        -33
Ireland                      2.08 (32)         1.37 (26)         -34          1.02 (15)        0.42 (11)         -59
Italy                        2.37 (615)        1.16 (420)        -51          1.45 (414)       0.67 (286)        -54
Netherlands                  2.08 (118)        0.96 (86)         -54          1.40 (82)         0.47 (56)        -66
Norway                       1.89 (37)         0.72 (21)         -62          1.23 (26)         0.51 (18)        -59
Portugal (1980-84)           0.94 (50)         0.68 (40)         -28         0.45 (29)          0.35 (25)        -22
Spain                        1.06 (155)        0.82 (178)        -23         0.55 (91)         0.45 (121)        -18
Sweden                       1.97 (88)         0.59 (38)         -70          1.14 (55)        0.38 (29)         -67
Switzerland                  1.90 (53)         1.16 (51)         -39          1.46 (45)        0.70 (39)         -52
United Kingdom               1.99 (570)        0.85 (299)        -57          1.07 (358)        0.49 (213)       -54
Eastern Europe

Bulgaria (1965-69)           1.45 (68)         1.02 (57)         - 30        0.74 (36)         0.53 (30)         -28
Czechoslovakia               2.75 (192)        1.54 (133)        -44          1.49 (116)        0.84 (98)        -44
E. Germany (1975-79)         1.39 (132)        1.33 (127)         -4         0.72 (93)         0.81 (110)        +13
Hungary (1970-74)            1.06 (65)         1.25 (80)         + 18        0.58 (41)         0.75 (64)         +29
Poland (1960-64)             1.62 (312)        1.56 (309)         -4         0.66 (144)         0.69 (166)        +5
Romania (1965-69)            1.25 (116)        1.15 (138)b        -          0.53 (63)         0.57 (73)b         +8
Yugoslavia (1960-64)         1.14 (100)        1.53 (193)        +34         0.64 (64)          0.84 (121)       -31

aAge-standardised rates on the world standard population. b1980-84.

2b

co

0)1
0)

E
0
0
0

Co
0)

0)
02

- 5

E

o  4-

6  3.

,_1
0)2

a1 .
0)0
0

i I I I a I a

- --*- 0

I          I    I    I

I

55r

730   C. LA VECCHIA et al.

Table II Death certification ratesal/100,000 people aged 0-14 (and numbers of deaths) from Hodgkin's disease in selected European countries,

1955-59 and 1985-88

Males                                         Females

1955-59           1985-88                      1955-59           1985-88

Rate              Rate         Percent         Rate              Rate         Percent

(No. of deaths    (No. of deaths  difference   (No. of deaths    (No. of deaths  difference
Country (year)              per year)         per year)      in rates      per year)         per year)      in rates
Western Europe

Austria                      0.44 (3)         0.04 (.)c        -91          0.24 (2)          0.04 (.)        -83
Belgium                      0.31 (3)         0.00 (0)        -100          0.15 (2)          0.00 (0)       -100
Denmark                      0.30 (2)         0.04 (.)         -87          0.03 (.)          0.00 (0)       - 100
Finland                      0.28 (2)         0.00 (0)        - 100        0.03 (.)           0.00 (0)       - 100
France                       0.30 (18)        0.02 (1)         -93          0.12 (7)          0.01 (1)        -92
W. Germany                   0.34 (20)        0.05 (3)         -85          0.16 (9)          0.01 (1)        -94
Greece (1965-69)             0.51 (6)         0.03 (.)         -94          0.14 (2)          0.03 (.)        -79
Ireland                      0.26 (1)         0.17 (1)         -35          0.30 (1)          0.00 (0)       -100
Italy                        0.62 (40)        0.05 (4)         -92          0.24 (15)         0.04 (3)        -83
Netherlands                  0.19 (3)         0.02 (.)          -89         0.12 (2)          0.00 (0)       -100
Norway                       0.20 (1)         0.00 (0)        - 100         0.13 (1)          0.00 (0)       - 100
Portugal (1980-84)           0.12 (2)         0.15 (2)         +25          0.01 (.)          0.00 (0)       - 100
Spain                        0.30 (13)        0.03 (2)         -90          0.16 (6)          0.04 (2)        -75
Sweden                       0.22 (2)         0.00 (0)        - 100         0.02 (.)          0.00 (0)       -100
Switzerland                  0.14 (1)         0.07 (.)         -50          0.06 (.)          0.00 (0)       - 100
United Kingdom               0.22 (14)        0.03 (2)         -86          0.10 (6)          0.03 (2)        -70
Eastern Europe

Bulgaria (1965-69)           0.30 (3)         0.17 (2)         -43          0.18 (2)         0.08 (1)         -56
Czechoslovakia               0.72 (14)        0.17 (4)         -76          0.23 (4)         0.04 (1)         -83
E. Germany (1975-79)         0.08 (2)         0.06 (1)         -25          0.04 (1)         0.05 (1)         +25
Hungary (1970-74)            0.21 (2)         0.14 (2)         -33          0.09 (1)         0.04 (.)         -56
Poland (1960-64)             0.44 (31)        0.08 (4)         -82          0.14 (10)        0.04 (2)         -71
Romania (1965-69)            0.47 (12)        0.35 (ll)b       -26          0.03 (1)         0.09 (3)b       +200
Yugoslavia (1960-64)         0.27 (8)         0.32 (9)         + 19         0.07 (2)         0.22 (6)        +214

aAge-standardised rates on the world standard population. b198084. cLess than one death per year.

Table III Death certification ratesa/100,000 people aged 65 or over (and numbers of deaths) from Hodgkin's disease in selected European

countries, 1955-59 and 1985-88

Males                                           Females

1955-59           1985-88                       1955-59           1985-88

Rate              Rate          Percent         Rate               Rate         Percent

(No. of deaths    (No. of deaths   difference   (No. of deaths     (No. of deaths   difference
Country (year)               per year)         per year)       in rates      per year)          per year)      in rates
Western Europe

Austria                      7.54 (25)         4.55 (19)         -40         4.68 (23)          3.30 (28)        -30
Belgium                      5.91 (27)         5.85 (17)          - 1        2.47 (15)         3.65 (15)         +48
Denmark                      5.22 (11)         3.66 (13)         -30         3.41 (8)           1.67 (9)         -51
Finland                      4.24 (5)          6.72 (15)         +59         2.74 (5)          2.21 (11)         -19
France                       3.51 (68)         2.85 (92)         - 19        1.87 (59)          1.17 (67)        -37
W. Germany                   4.75 (105)        4.40 (150)         -7         2.74 (82)         2.51 (175)         -8
Greece (1965-69)             6.74 (25)         6.02 (37)         - 11        2.77 (13)         2.45 (18)         - 12
Ireland                      3.48 (5)          5.81 (10)         +67         2.25 (3)          3.02 (7)          +34
Italy                        5.85 (112)        4.91 (155)        -16         3.73 (94)         2.71 (127)        -27
Netherlands                  4.03 (19)         3.92 (24)          -3         2.54 (13)         2.41 (31)          -5
Norway                       5.49 (9)          3.75 (10)         -32         3.69 (7)          2.50 (11)         -32
Portugal (1980-84)           3.06 (14)         2.38 (13)         -40          1.62 (11)         1.02 (8)         -37
Spain                        2.80 (28)         3.02 (54)          +8         1.68 (24)          1.97 (56)        + 17
Sweden                       7.18 (29)         2.22 (16)         -69         4.16 (19)          1.99 (18)        -52
Switzerland                  3.77 (8)          5.67 (23)         +50         3.46 (10)         3.70 (22)          +7
United Kingdom               4.66 (111)        2.41 (87)         -48         2.95 (105)         1.64 (96)        -44
Eastern Europe

Bulgaria (1965-69)           4.63 (16)         2.32 (11)         -50         2.49 (10)          1.36 (8)         -45
Czechoslovakia               7.70 (36)         6.31 (42)         -18         4.56 (30)          3.97 (47)        - 13
E. Germany (1975-79)         4.80 (47)         5.73 (43)         + 19        2.45 (42)          3.47 (57)        +42
Hungary (1970-74)            3.95 (21)         4.99 (26)         +26          1.90 (14)         3.73 (32)        +96
Poland (1960-64)             3.00 (30)         4.98 (66)         +66          1.43 (23)         2.37 (54)        +66
Romania (1965-69)            2.75 (21)         2.43 (23)b        - 12        1.62 (16)          1.07 (14)b       -34
Yugoslavia (1960-64)         2.83 (14)         6.49 (55)         + 129        1.62 (12)         2.70 (33)        +67

aAge-standardised rates on the world standard population. bl980-84.

younger (under 35) and middle age (35 to 64 years), although
generally larger, in proportional terms, in the young, but in
absolute terms in middle age. Even more substantial declines
were observed in childhood (Table II), too, and, in larger
countries, some fall was discernible even in the more confus-
ing pattern of trends in the oldest age group (over age 65)
(Table III and Figure 3). The declines were steady and

persistent up to the most recent calendar period, with no
evidence of levelling of rates.

The pattern of trends in mortality from Hodgkin's disease
was largely different in the seven eastern and central
European countries. Among them, some fall was observed
only in Bulgaria (approximately 30%) and Czechoslovakia
(44%), although there was some evidence of levelling of rates

HODGKIN'S DISEASE MORTALITY IN EUROPE  731

FRANCE

5 60

65  70   75  80  85   90
Calendar years

ITALY

A

5  60   65  70   75  80   85  90

Calendar years

PORTUGAL

5  60   65  70   75  80   85  90

Calendar years

C
0

o 12-

0. 10-

0

a- 8

0
0

o   6

0

4-
+   2-

C0
CU

* 12.

X.10-
0

8-

Qv

C'- 6-

Cl)

4

CD

2-

0

tJ

0

'- 12-

0.

O  10-

o 8-
o    6-
u 4-

CU

' 2

0    O

5) c

WESTERN GERMANY

5 60 65    70   75  80  85

Calendar years

90

NETHERLANDS

I&_

5 60

65  70   75  80 85
Calendar years

90

SPAIN

_-*

55  60   65  70   75  80   85

Calendar years

90

UNI I tED KINGDOM

z          z      z      z I  I

55  60   65  70    i5  80      85  90

Calendar years

Figure 3 Trends in mortality from Hodgkin's disease in seven western and six eastern European countries from 1955 to 1988.
Age-standardised (world) rates in males (-A ) and females ( A-) aged 65 and over.

over more recent calendar periods. Other countries showed
no consistent pattern, and some of them (Hungary and
Yugoslavia) showed some increase. The overall mean differ-
ence of rates over the last few decades in the eastern block
countries were -5% for males and +2% for females. Only
in children before age 15 was some decline in rates evident in
Eastern Europe, too.

Figure 4 contrasts incidence and mortality rates for five
countries where national cancer incidence statistics have been
available since 1960 (Denmark, Finland, Norway, Scotland
and Sweden). In all countries, mortality declined while
incidence showed little systematic change, leading to a sub-
stantial widening of the incidence/mortality ratios from an
average of 1.35 in 1960 to 2.38 in 1980 (Table IV).

Discussion

This analysis of trends in Hodgkin's disease mortality in 23
European countries shows a clear divergence between consis-
tent declines over the last two decades in all western count-

ries, and no evidence of systematic falls in Eastern Europe.

It is unlikely that problems of diagnosis and certification
of Hodgkin's disease (particularly with respect to non-Hodg-
kin's lymphomas) (Gruffermann & Delzell, 1986; Glaser &
Swartz, 1990), which might have somewhat influenced the
rates, could explain the observed trends. The pathological
entity of Hodgkin's disease, with its pathognomonic Reed-
Sternberg cell, has been recognised for almost a century now
(Reed, 1902; Sternberg, 1893; Hellmann, 1991). In relation to
these potential problems, it is also reassuring that the pattern
of trends, in most countries, was similar at younger and
middle age, when diagnosis and certification of the disease
has long been more accurate (Doll & Peto, 1981).

A further support to the existence of a major role of
therapy, rather than diagnostic artifacts, on these trends
comes from a comparison of incidence and mortality rates
for European Community Members States in 1980-84, since
incidence rates were two to three times higher than the
corresponding mortality, the differences being greater in
younger ages (Jensen et al., 1990). In the early 1980s, the
differences between incidence and mortality were systematic-

3a     c

*' 12-

.3

10-

0.

Q

o> 6 -

- 45

en

0

B. 2 -

m   5
C

0

._o i )

0.

o   8

0

. 6'
0
0

4'

CU

4-  2

U     a

C
0

t4-      I ')

CU IL'

i- 10'
0
0.

0

o   8-

0

0   6
d

4'
C6U.  2-

41  0

WD   I

a

C
0

CU  12-

a10 -

8-
0

C)  8-

0

C,,

CO  2
o 0e

CvU'

X-  4 -

CU

0

i

I                                   I            I           I           I

I     I     I      I     I   -r----i

i

I            I            a             I             I            I             I

i

F         I        I         I

-..                                                   I             I             I

A Ilk. .-- - .1 . . I - - - . .

732    C. LA VECCHIA et al.

,^1  CZECHOSLOVAKIA

c
0
4--

Co

0

0.

0

0

0

6

Ov

co

Co

4-

Co

0)

55 60 65 70 75 80 85 90

Calendar years

12 -
10 -

8-
6
4.
2
0o

GDR

55 60 65 70 75 80 85 90

Calendar years

HUNGARY

5  60   65   70   75   80   85   90

Calendar years

ROMANIA

55 60

C
0
Co

0._

0

0
Co
Q
Co
o

Co

0

iu

C
0
Co

0._
-

10
0

0
0

CO

Co

Co

0
a)
c]

65  70   75  80  85 90
Calendar years

12-
10-
8-
6-

4-
2-

0-

POLAND

55  60  65 70    75  80  85  90

Calendar years

12-
10-
8-
6-
4-
2-

YUGOSLAVIA

55 60

65 70 75 80
Calendar years

85 90

ally smaller in areas covered by cancer registration from
Eastern Europe (Levi et al., 1989). Further, no major and
systematic change was observed in non-Hodgkin's lympho-
mas mortality over the same calendar period (unpublished
data from the WHO databank). Socio-demographic changes
may influence sub-type and age distribution of Hodgkin's
disease, since nodular sclerosis is more common at younger
age and among higher social classes (Glaser, 1990; Serraino
et al., 1991). This may well influence survival rates, but is
unlikely to largely explain the mortality trends observed.

Registered mortality trends were less consistent above age
65, but this is compatible with generalised decline in reli-
ability and validity of cancer death certification in the elderly
(Doll & Peto, 1981). In addition, experience from all coun-
tries indicates that the prognosis for elderly patients with
Hodgkin's disease remains poor, despite clear improvements
in therapy. Still, this is less important for Hodgkin's disease
than for epithelial cancers, since Hodgkin's disease incidence
does not rise with a power of age (Cook et al., 1969; Doll,
1971), and the proportion of cases in the elderly is conse-
quently smaller than for epithelial neoplasms.

The major determinant of the favourable trends in morta-
lity in Western Europe has been improved treatment of the
disease, as confirmed also by comparison of incidence and
mortality trends in selected areas. These clear successes not-
withstanding, the present data gives scope for a few critical
considerations. First, advancements in radiotherapy have
been available since the 1950s, and effective chemotherapy in
clinical series from research settings has been available since
the early 1960s (De Vita et al., 1965; Rosenberg, 1989). Only
from the late 1960s or early 1970s onwards, however, im-
provements became evident in national mortality data from
more advanced western European countries, and in a few
countries rates started to fall only during the late 1970s. This
is consisted with a modelling of survival data from the US

Table IV Incidence/mortality ratios for Hodgkin's disease in selected

European countries, circa 1960 and 1980

Incidence/mortality ratios for:

Males, circa           Females, circa

Country         1960        1980         1960        1980
Denmark          1.28        2.36        1.27        2.29
Finland          1.32        1.43        1.63        2.43
Norway           1.35        2.17        1.31        2.67
Sweden           1.45        2.30        1.58        2.50
Scotland         1.05        3.00        1.22        2.60

National Cancer Institute's Surveillance, Epidemiology, and
End Results (SEER) program, which showed that dissemina-
tion of improved survival took approximately 11 years for
Hodgkin's disease (but only three for testicular cancer; Feuer
et al., 1991). This longer delay was attributed to the difficulty
in replicating the very positive results of the original MOPP
trial, of staging Hodgkin's disease, and to the fact that
treatment of Hodgkin's disease is less frequently referred to
specialist centres.

Thus, the most likely interpretation of this pattern is that
the utilisation of effective treatments of Hodgkin's disease,
which have been established since over two decades now, has
been different in various parts in Europe. In absolute terms,
the reductions in Hodgkin's disease mortality in Western
Europe correspond to the avoidance of over 3,000 deaths per
year, which is the major established therapeutic advancement
for any cancer site (Cairns & Boyle, 1983; Boyle et al., 1988;
La Vecchia et al., 1989). This figure could well approach
4000 deaths avoided per year if the larger advancements
observed in a few Scandinavian countries had been registered
in other western European countries, too.

3b

c
0

11

M

0 1
ol

0.
0
0

Co
co

CD

n

aC

0

10-
8-
6-
4-

2 -

C
0

Co

0._

0

0.:

0

0

0

6

0

Co

Co
Co
Co

CU

0)

a1)

co

0._

-

0.
0

0

0

0

0

Co
Co

Co

Co
co
n
4)
4)

CD

12 -

10

8-
6-
4-
25

0r

5'

12-
10

8
6
4'
2

U

i

9            I                                         I           I

n)

i

i                                     - v         .      ---I        I

Ak

I                                 I       I

&--?

Ac A,

i       I   I    I   a

v-

r---r

HODGKIN'S DISEASE MORTALITY IN EUROPE            733

SCOTLAND
5

o 4

63                   U

0)

lw  1.1A                  Z~

0

1960  1965  1970 1975  1980

Calendar period

5           DENMARK                        5           FINLAND

0                                        0

O~~~~~__ 4                                                            u

(D 21                                              ~

2u1

0   -                                      0

1960  1965  1970 1975  1980                1960  1965  1970 1975 1980

Calendar period                           Calendar period

5           NORWAY                         s          SWEDEN

0 4                                        0 4

4---                                   4 L
0                                        0

2                                          2 3     -----

A ----       ,        ___             C2

0                                          0

1960 1965 1970   1975  1980                1960  1965 1970  1975 1980

Calendar period                            Calendar period

u- INCIDENCE MALES     -o- MORTALITY MALES

A- INCIDENCE FEMALES -&- MORTALITY FEMALES

Figure 4 Comparison in trends in mortality and incidence rates in five European countries, circa 1960 to 1980.

Even within Western Europe, there is certainly scope for
future progress, as indicated by the different patterns of
trends in various countries and by the observation that rates
are still downwards in most recent periods in most areas.

A major indication emerging from this analysis, moreover,
is on the importance and urgency of improving the avail-
ability of currently defined knowledge and resources for the
treatment of Hodgkin's disease, as well as of other types of
curable cancers (Boyle et al., 1990), in Central and Eastern
Europe.

Further study in these countries will determine whether the
major problem relates to a failure to refer patients for treat-

ment at specialised cancer centres, where the results of treat-
ment for curable neoplasms such as Hodgkin's disease may
be expected to be superior to those in non-specialist centres.
An alternative explanation could be the lack of availability of
certain cytotoxic agents, and this would be particularly
regrettable, as the drugs involved have been widely used for
several years.

The contribution of the Swiss League against cancer is gratefully
acknowledged. We wish to thank Mr Vincenzo and Felice De Ceglie
for graphical assistance.

References

BONADONNA, G. (1982). Chemotherapy strategies to improve the

control of Hodgkin's disease: the Richard and Hinda Rosenthal
Foundation Award Lecture. Cancer Res., 42, 4309.

BOYLE, P., SOUKOP, M., SCULLY, C. & 4 others (1988). Improving

prognosis of Hodgkin's disease in Scotland. Eur. J. Cancer Clin.
Oncol., 24, 229.

BOYLE, P., MAISONNEUVE, P. & KAYE, S.B. (1990). Therapy for

testicular cancer in Central and Eastern Europe. Lancet, i, 1033.
CAIRNS, J. & BOYLE, P. (1983). Cancer chemotherapy. Science, 22,

252.

COOK, P.J., DOLL, R. & FELLINGHAM, S.A. (1969). A mathematical

model for the age distribution of cancer in man. Int. J. Cancer, 4,
93.

DE VITA, V.T., MOXLEY, J.M., BRACE, K. & FREI, E. (1965). Inten-

sive combination chemotherapy and X-irradiation in treatment of
Hodgkin's disease. Proc. Am. Assoc. Cancer Res., 6, 15.

DE VITA, V.T., SERPICK, A. & CARBONE, P.P. (1970). Combination

chemotherapy in treatment of advanced Hodgkin's disease. Ann.
Intern. Med., 73, 881.

734    C. 'LA VECCHIA et al.

DE VITA, V.T., SIMON, R.M., HUBBARD, S.M. & 6 others (1980).

Curability of advanced Hodgkin's disease with chemotherapy.
Long tenn follow-up of M.O.P.P. treated patients at the National
Cancer Institute. Ann. Intern. Med., 92, 587.

DOLL, R. (1971). The age distribution of cancer: implications for

models of carcinogenesis. J. R. Stat. Soc., Series A, 134, 133.

DOLL, R., MUIR, C.S. & WATERHOUSE, J.A.H. (1970) (eds). Cancer

Incidence in Five Continents, Vol. II. Springer-Verlag: Berlin.

DOLL, R., PAYNE, P. & WATERHOUSE, J.A.H. (1966) (eds). Cancer

Incidence in Five Continents, Vol. I. Springer-Verlag: Berlin.

DOLL, R. & PETO, R. (1981). The causes of cancer. Quantitative

estimates of avoidable risks of cancer in the United States today.
J. Nati Cancer Inst., 66, 1191.

DOLL, R. & SMITH, P. (1982). Age-Standardised Incidence Rates. In

Cancer Incidence in Five Continents, Vol. IV, Waterhouse, J.A.H.,
Muir, C.S., Shanmugaratnam, K. & Powell, J. (eds). IARC
Scient. Publ. No. 88. IARC: Lyon.

FEUER, E.J., KESSLER, L.G., BAKER, S.G., TRIOLO, H.E & GREEN,

D.T. (1991). The impact of breakthrough clinical trials on survival
in population based tumor registries. J. Clin. Epidemiol., 44, 141.
GLASER, S.L. & SWARTZ, W.G. (1990). Time trends in Hodgkin's

disease incidence. The role of diagnostic accuracy. Cancer, 66,
2196.

GLASER, S.L. (1990). Hodgkin's disease in black populations: a

review of the epidemiologic literature. Sem. Oncol., 17, 643.

GRUFFERMAN, S. & DELZELL, E. (1984). Epidemiology of Hodg-

kin's disease. Epidemiol. Rev., 6, 76.

HELLMANN, S. (1991). Thomas Hodgkin and Hodgkin's disease.

Two paradigms appropriate to medicine today. JAMA, 265,
1007.

JENSEN, O.M., ESTEVE, J., M0LLER, H. & RENARD, H. (1990).

Cancer in the European Community and its Member States. Eur.
J. Cancer, 26, 1167.

LA VECCHIA, C., BOYLE, P., CISLAGHI, C., DECARLI, A. & NEGRI,

E. (1989). Descriptive epidemiology of Hodgkin's disease in Italy.
Tumori, 75, 401.

LEVI, F., MAISONNEUVE, P., FILIBERTI, R., LA VECCHIA, C. &

BOYLE, P. (1989). Cancer incidence and mortality in Europe. Soz.
Praeventivmed., 34 (Suppl. 2), S3.

MUIR, C.S., WATERHOUSE, J.A.H., POWELL, J., MACK, T. &

WHELAN, S. (1988) (eds). Cancer Incidence in Five Continents,
Vol. V. IARC Scient Publ No. 88. IARC: Lyon.

REED, D.M. (1902). On the pathological changes in Hodgkin's

disease with special reference to its relation to tuberculosis. John
Hopkin's Hosp. Rep., 10, 133.

ROSENBERG, S.A. (1989). Hodgkin's disease: challenges for the

future. Cancer Res., 49, 767.

SERRAINO, D., FRANCESCHI, S., TALAMINI, R. & 4 others (1991).

Socio-economic indicators, infectious diseases and Hodgkin's
disease. Int. J. Cancer, 47, 352.

STERNBERG, C. (1893). Uber eine Eigenartige unter dem Bilde der

Pseudoleukaemie verlaufende Tuberkulose des lymphatischen
Apparates. Zeitschr. Heilkunder, 19, 21.

WATERHOUSE, I.A.H., MUIR, C.S., CORREA, P. & POWELL, J. (1976)

(eds). Cancer Incidence in Five Continents, Vol III. IARC Scient
Publ No 26. IARC: Lyon.

WATERHOUSE, J.A.H., MUIR, C.S., SHANMUGARATNAM, K. &

POWELL, J. (1982) (eds). Cancer Incidence in Five Continents, Vol.
IV. IARC Scient Publ No 46. IARC: Lyon.

				


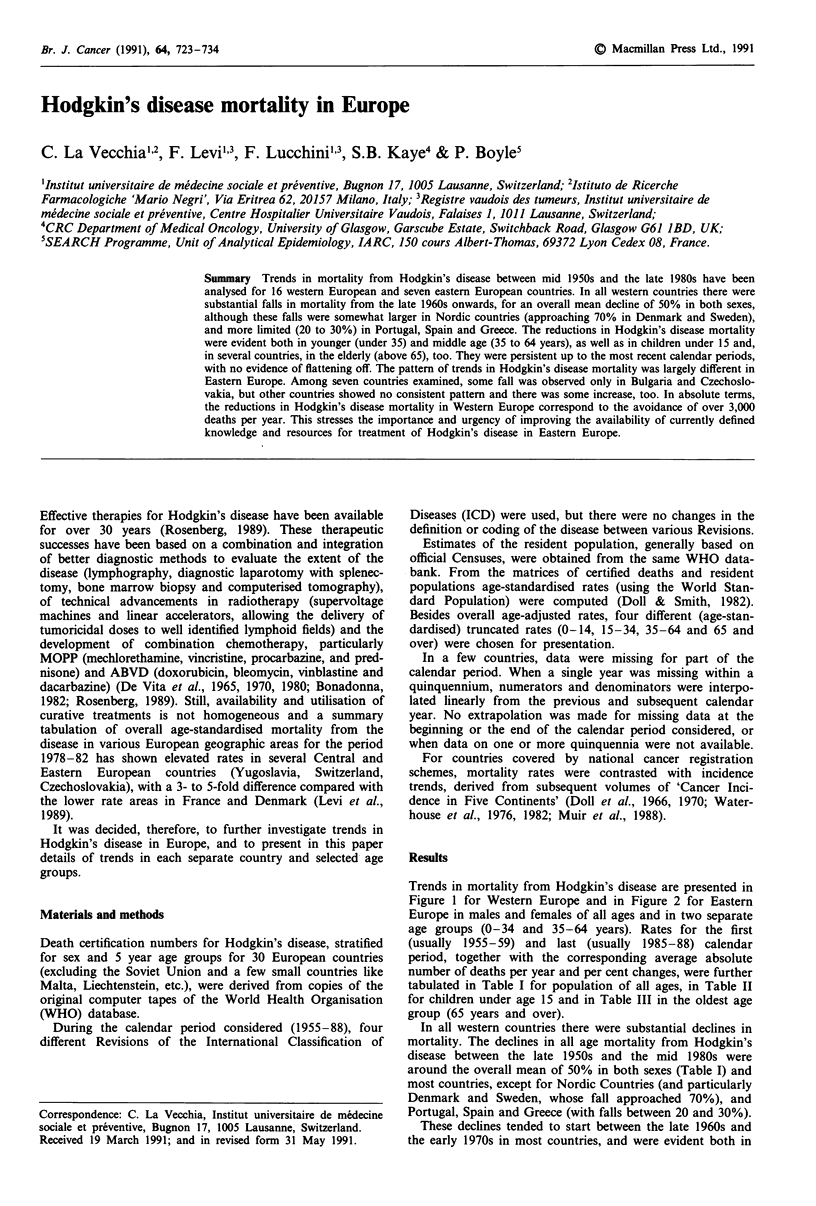

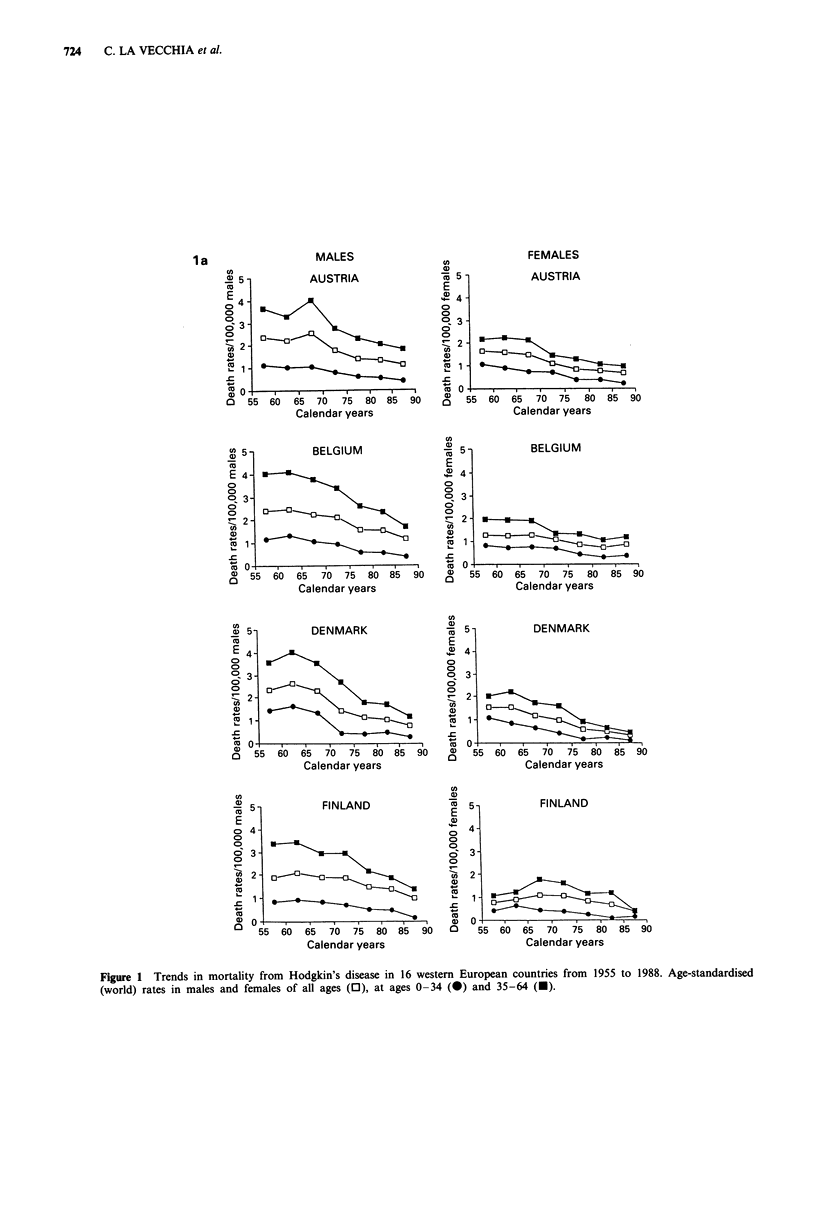

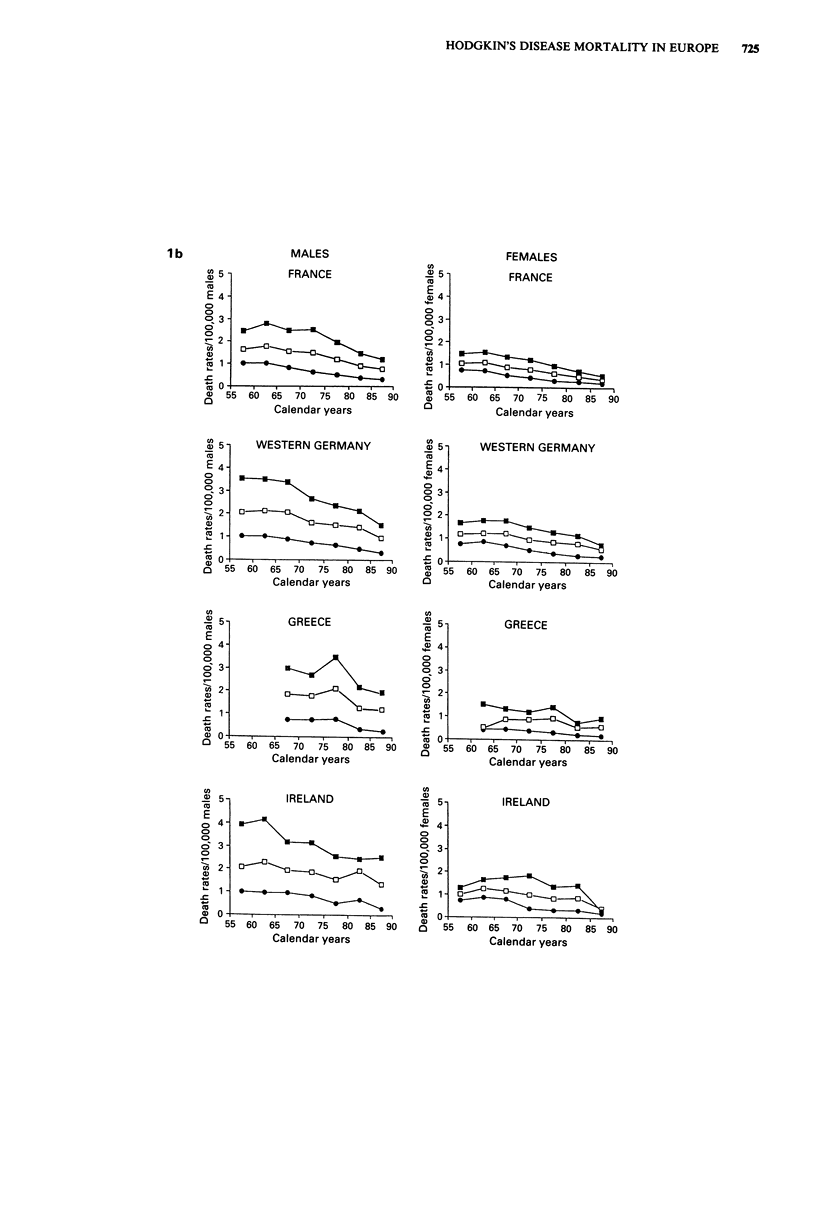

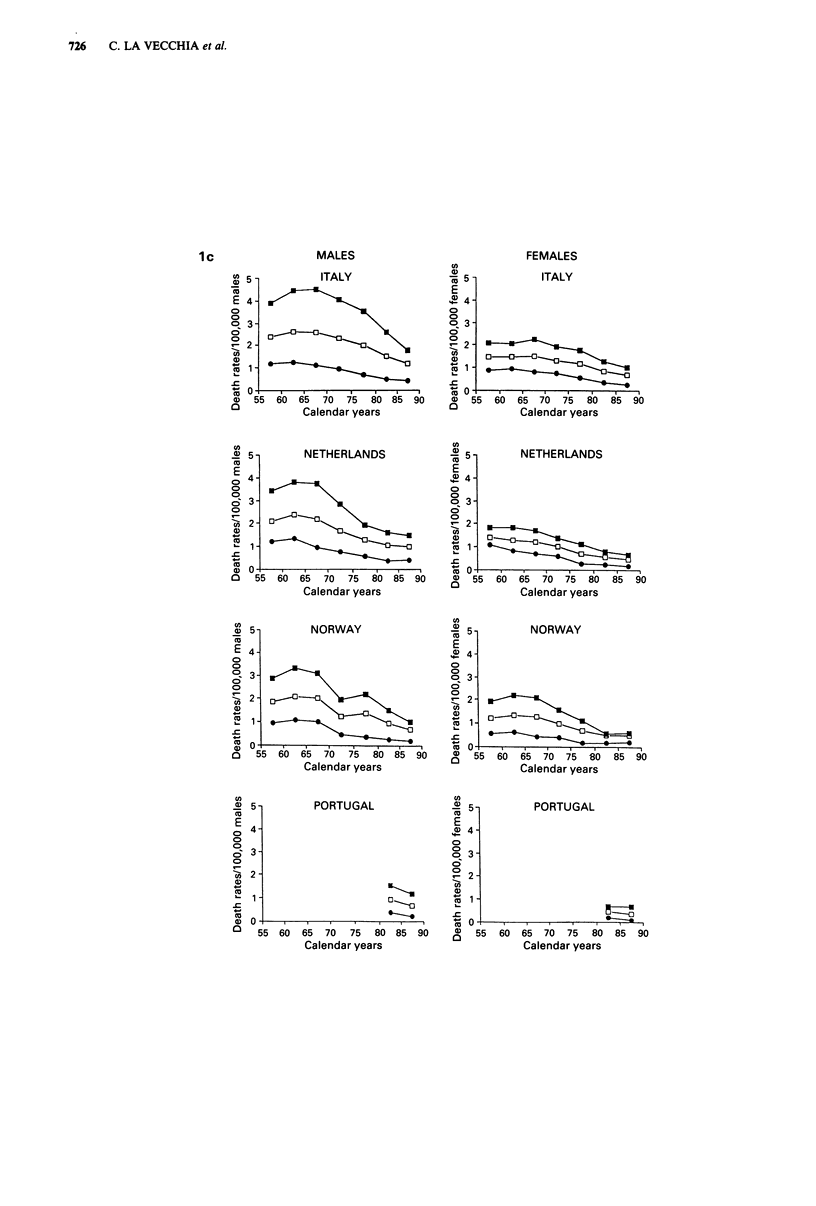

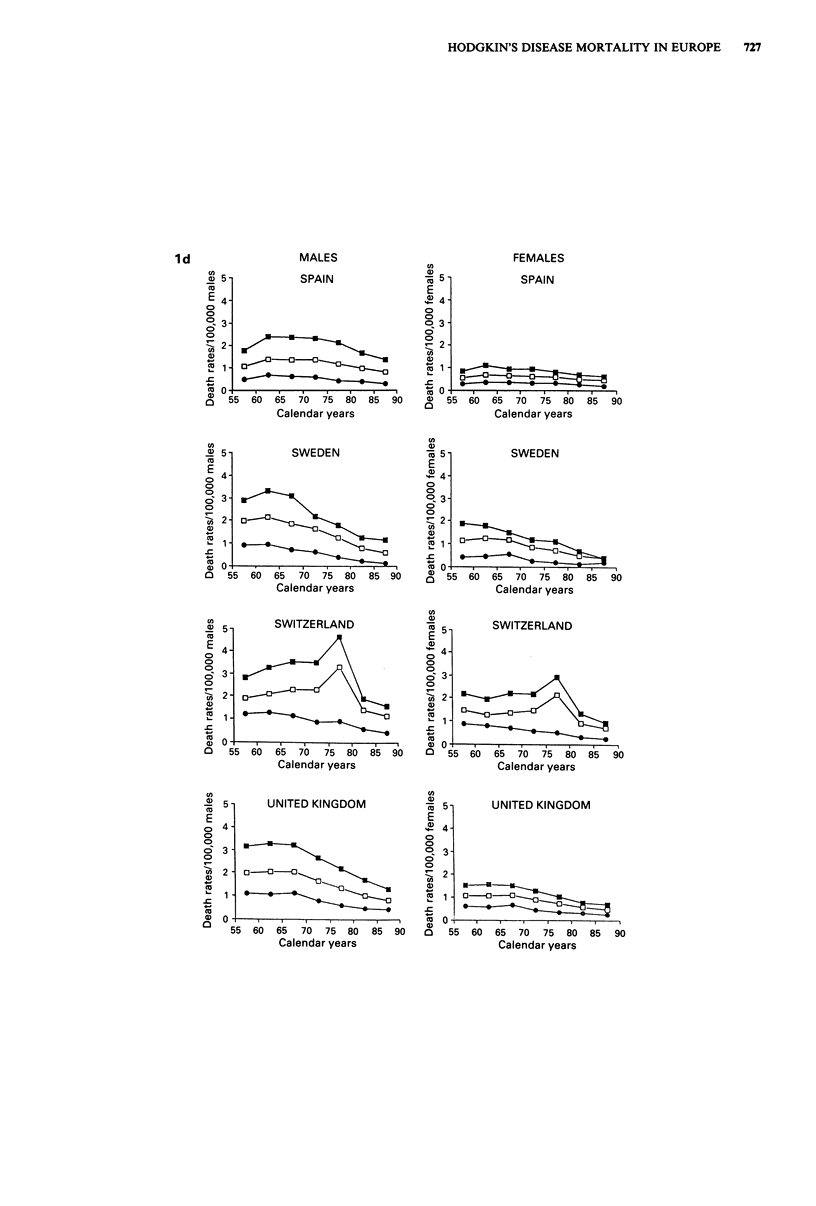

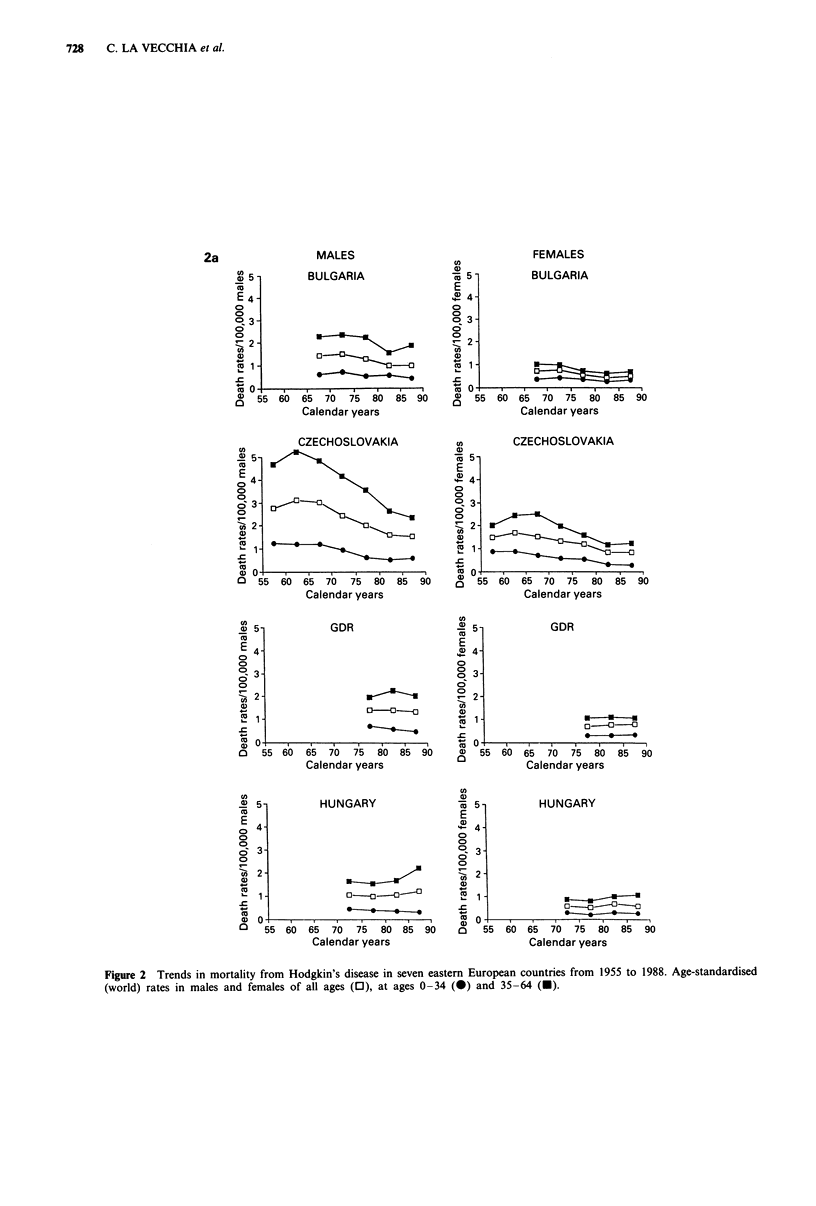

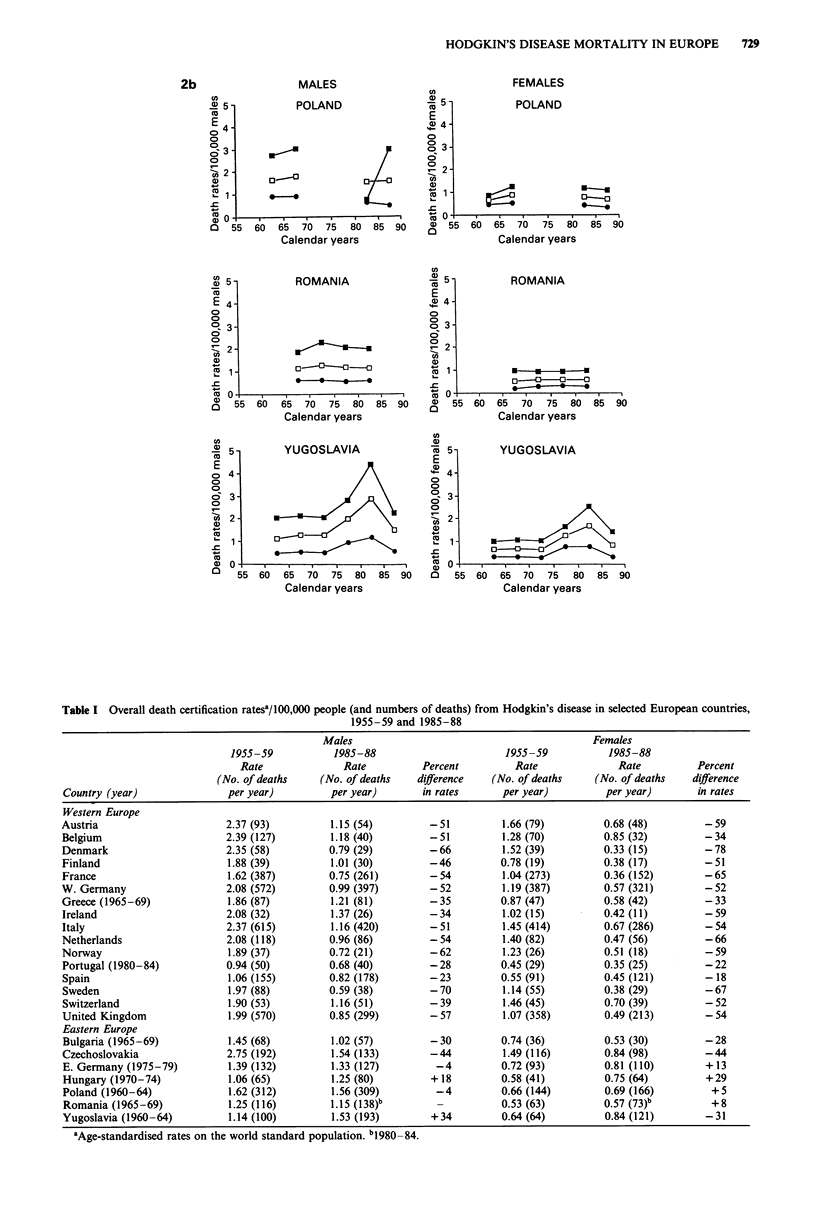

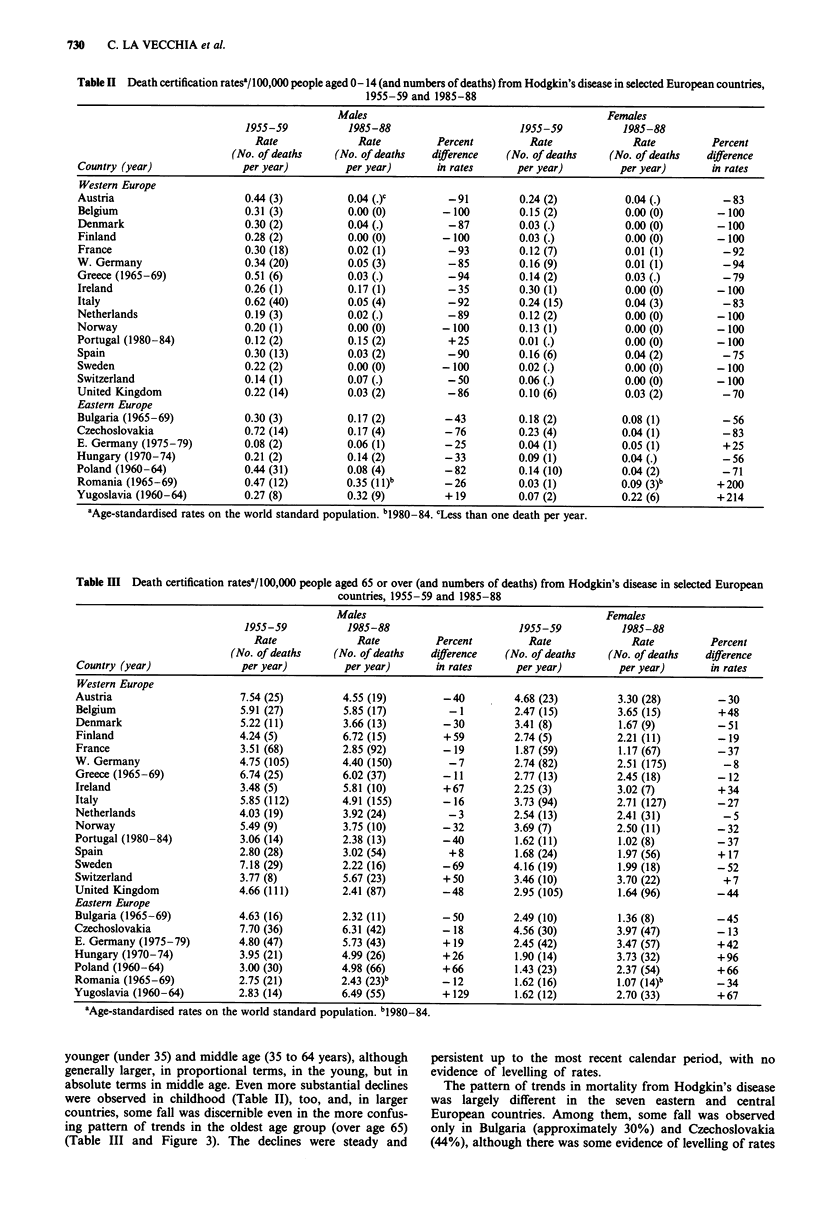

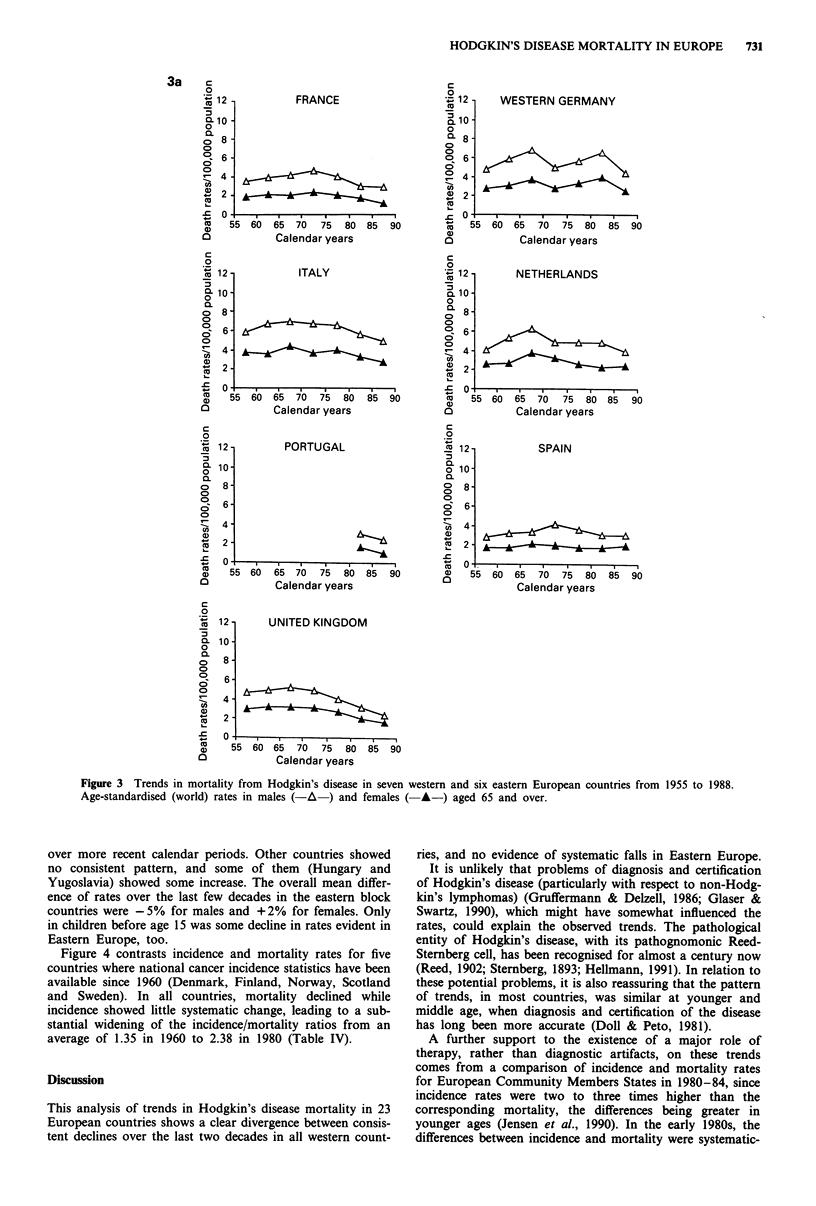

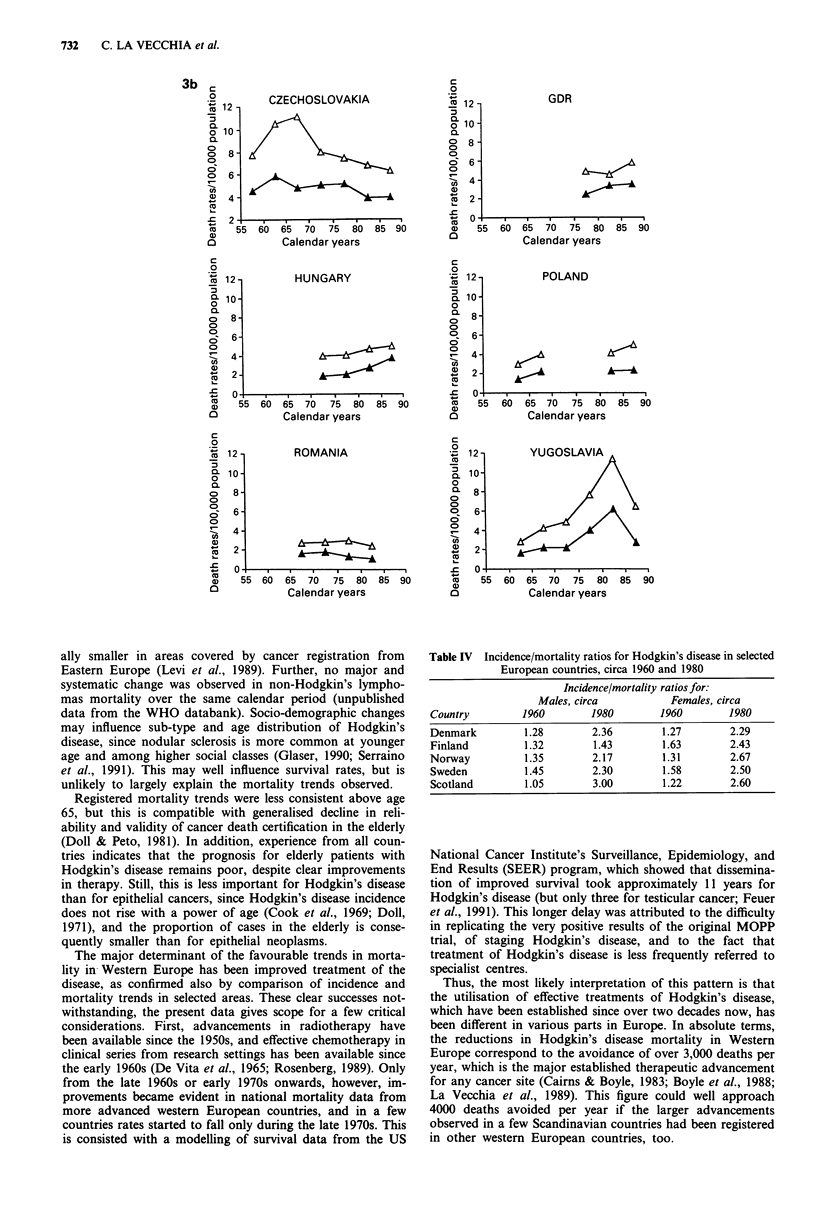

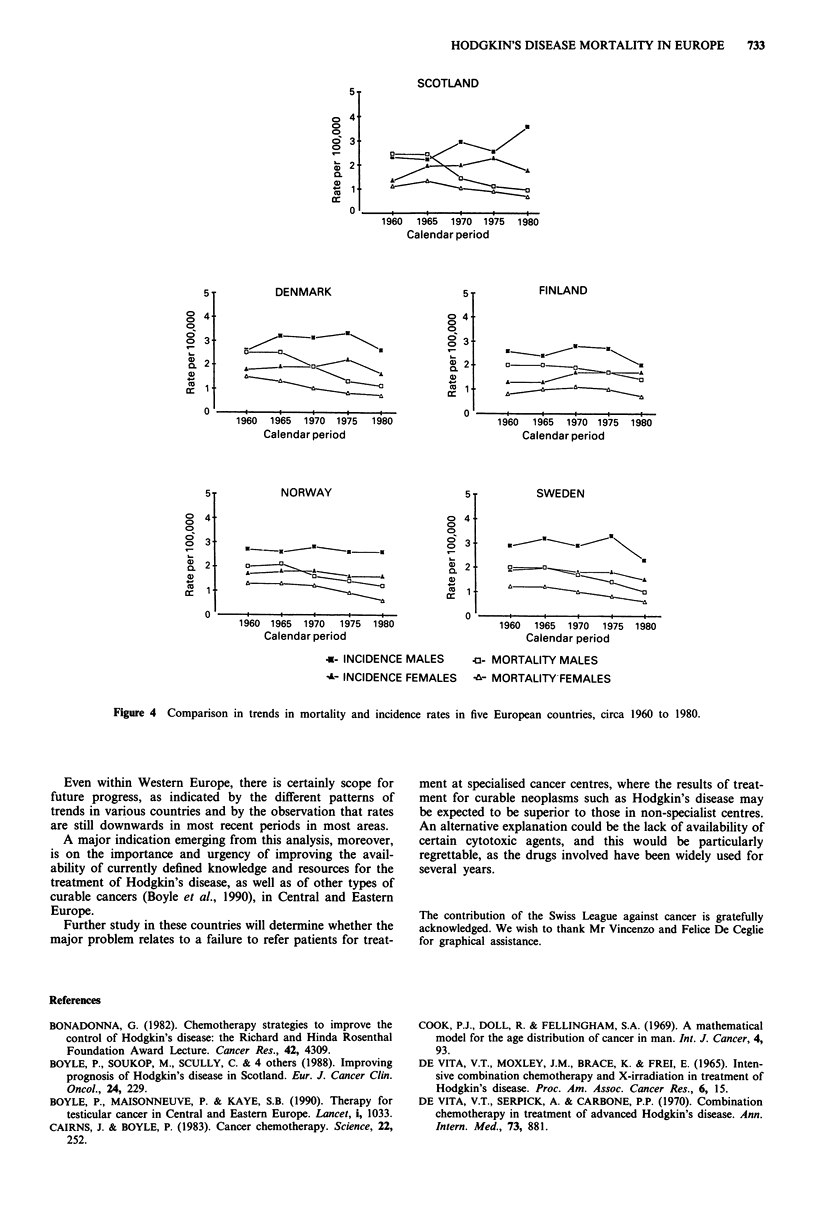

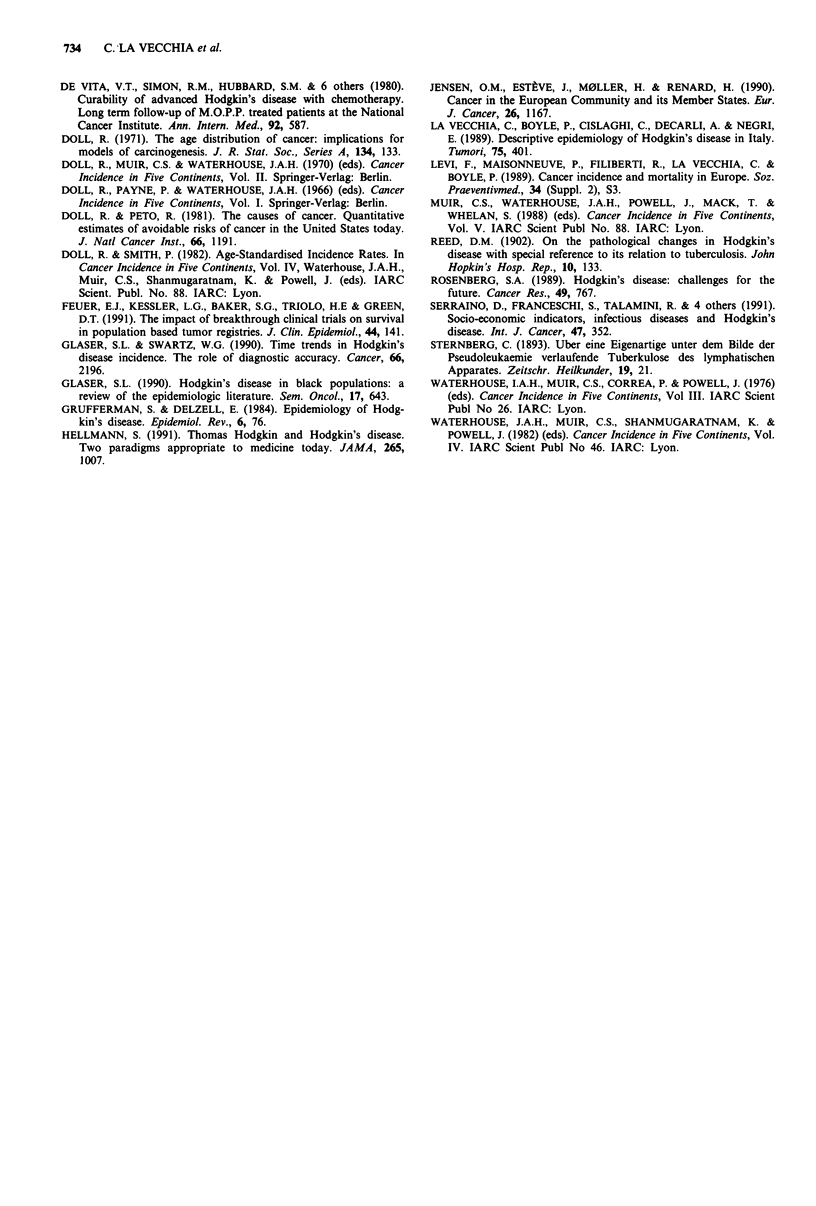

